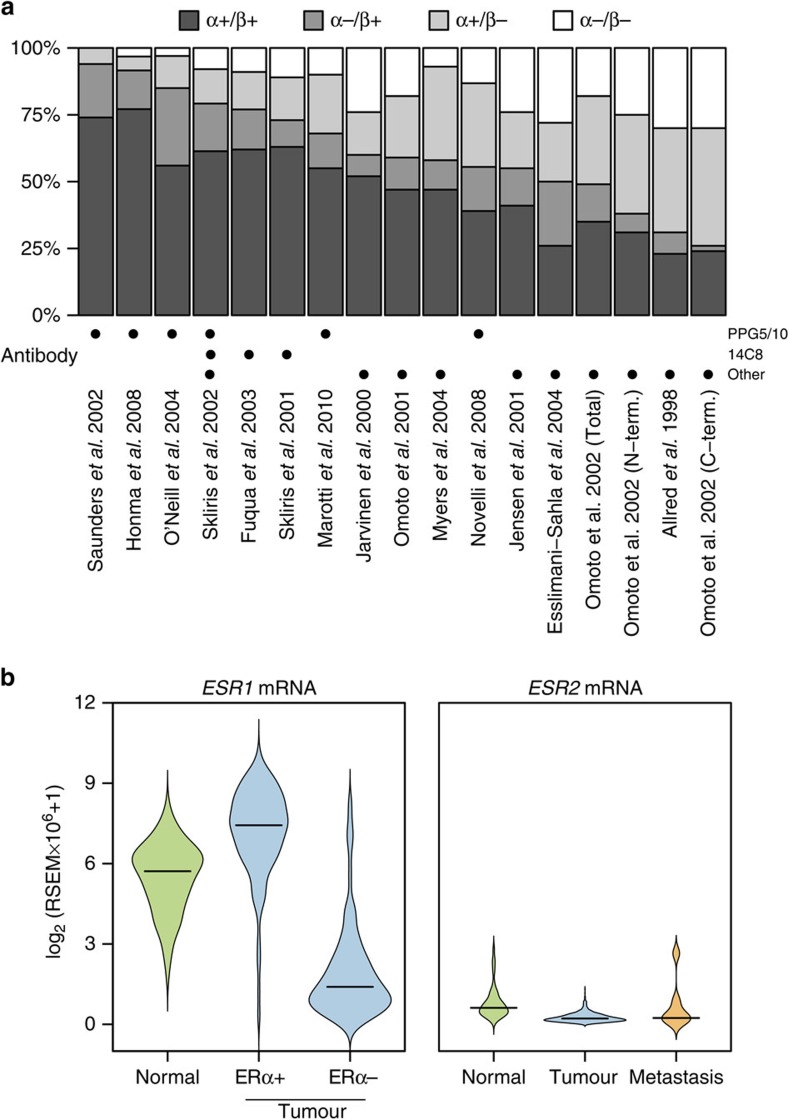# Corrigendum: Insufficient antibody validation challenges oestrogen receptor beta research

**DOI:** 10.1038/ncomms16164

**Published:** 2017-11-29

**Authors:** Sandra Andersson, Mårten Sundberg, Nusa Pristovsek, Ahmed Ibrahim, Philip Jonsson, Borbala Katona, Carl-Magnus Clausson, Agata Zieba, Margareta Ramström, Ola Söderberg, Cecilia Williams, Anna Asplund

Nature Communications 8: Article number: 15840; DOI: 10.1038/ncomms15840 (2017); Published 06 15 2017; Updated 11 29 2017

In this Article, two papers are mistakenly listed as having made use of the antibody 14C8 instead of the antibody PPG5/10. In the Discussion section, ref. 45 is incorrectly cited as having shown that the antibody 14C8 works well, and in Fig. 5a, Saunders *et al.* 2000 is incorrectly depicted as using the 14C8 antibody. Both these papers used antibody PPG5/10 and neither paper includes experiments using 14C8. A corrected version of Fig. 5 appears below as Fig. 1.

## Figures and Tables

**Figure 1:**