# Supplementary material for: Insufficient antibody validation challenges oestrogen receptor beta research
Source: Nat Commun. 2017 Jun 15;8:15840. doi: 10.1038/ncomms15840 (PMC5501969; doi:10.1038/ncomms15840)
Supplement: Supplementary Information — Supplementary Figures, Supplementary Tables and Supplementary References [file ncomms15840-s1.pdf]

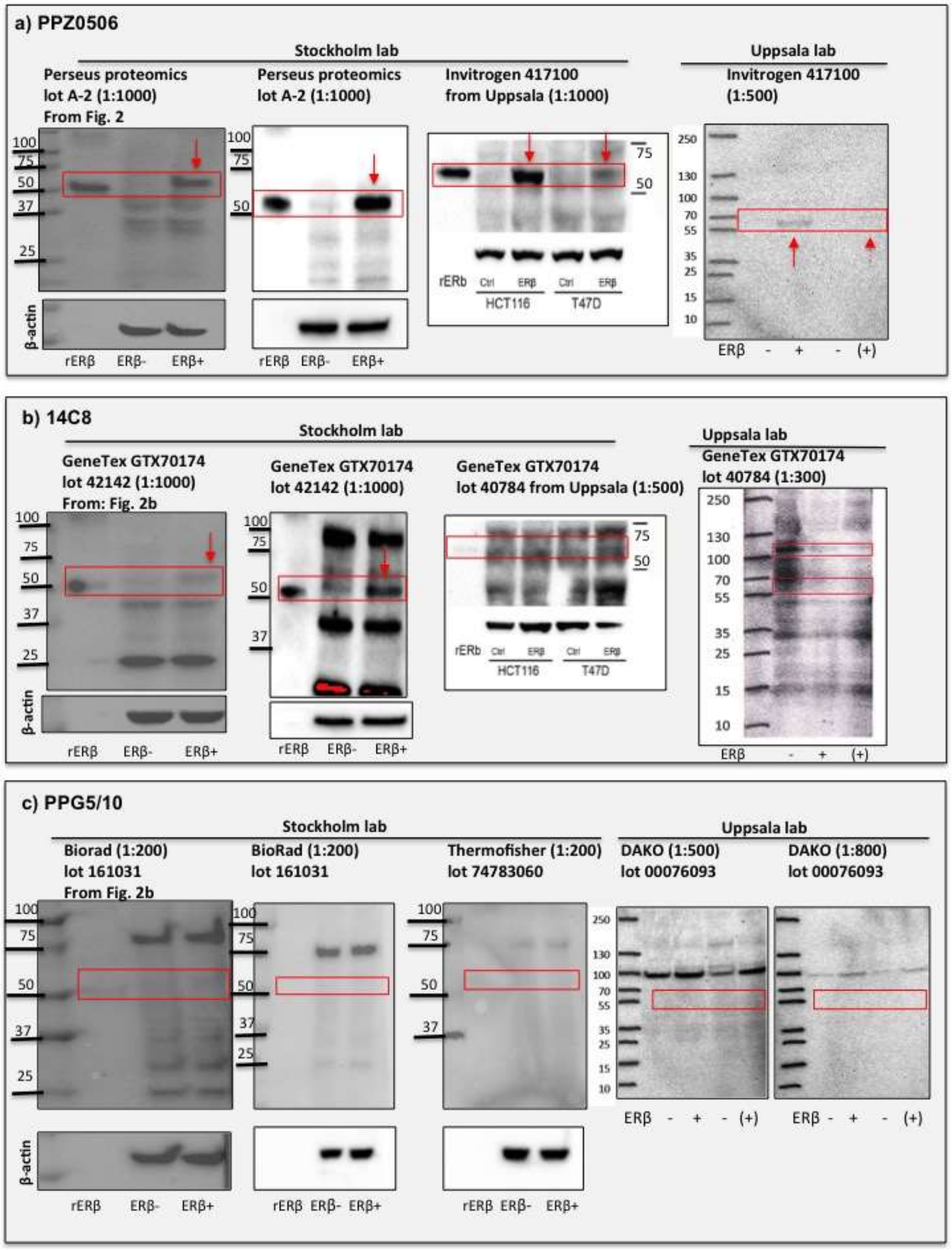

Supplementary Figure 1. Western blotting with ERβ antibodies
Full blots corresponding to Fig. 2, along with replicated experiments at different time points, different batches, and different labs. ERβ-positive (HCT116-ERβ), weakly ERβ-positive T47D-ERβ, and ERβ-negative (HCT116-mock, T47D-mock) control cells, recombinant ERβ (rERβ), lot number, dilution, b-actin loading control, and size marker as indicated. Red box indicates expected band location for ERβ (59-60 kDa), red arrow points out specific bands. **a)** Antibody PPZ0506 generates reproducible results; **b)** antibody 14C8 show variable reproducibility and looses affinity for ERβ after some storage. Both stored and new antibody show unspecific bands; **c)** PPG5/10 does not detect ERβ, but strongly stains unspecific bands of approximately 75-100 kDa in both positive and negative controls.

TISSUE	RNA (FPKM)	IHC 1D5
Endometrium	161	
Fallopian tube	142	
Smooth muscle	114	
Breast	49	
Ovary	12	
Prostate	8	
Liver	9	
Thyroid gland	6	
Gallbladder	3	
Testis	3	
Tonsil	3	
Kidney	2	
Urinary bladder	2	
Skeletal muscle	2	
Lymph node	2	
Adrenal gland	2	
Lung	2	
Esophagus	1	
Stomach	1	
Appendix	1	
Colon	1	
Rectum	1	
Skin	1	
Spleen	1	
Heart muscle	1	
Pancreas	0	
Salivary gland	0	
Duodenum	0	
Small intestine	0	
Placenta	0	
Bone marrow	0	
Cerebral cortex	0	

Supplementary Figure 2. IHC with ERα antibody 1D5 shows congruency with ERα transcript levels in large panel of tissues

Average ERα transcript levels (FPKM) measured using RNA-seq in triplicate samples of 32 normal tissues in the Human Protein Atlas tissue panel,[1] displayed alongside annotated IHC positivity generated with antibody 1D5 (1:150). Annotated IHC positivity is dichotomized into grades 1-3, represented by colors (1-light brown, 2-medium brown, 3-dark brown).

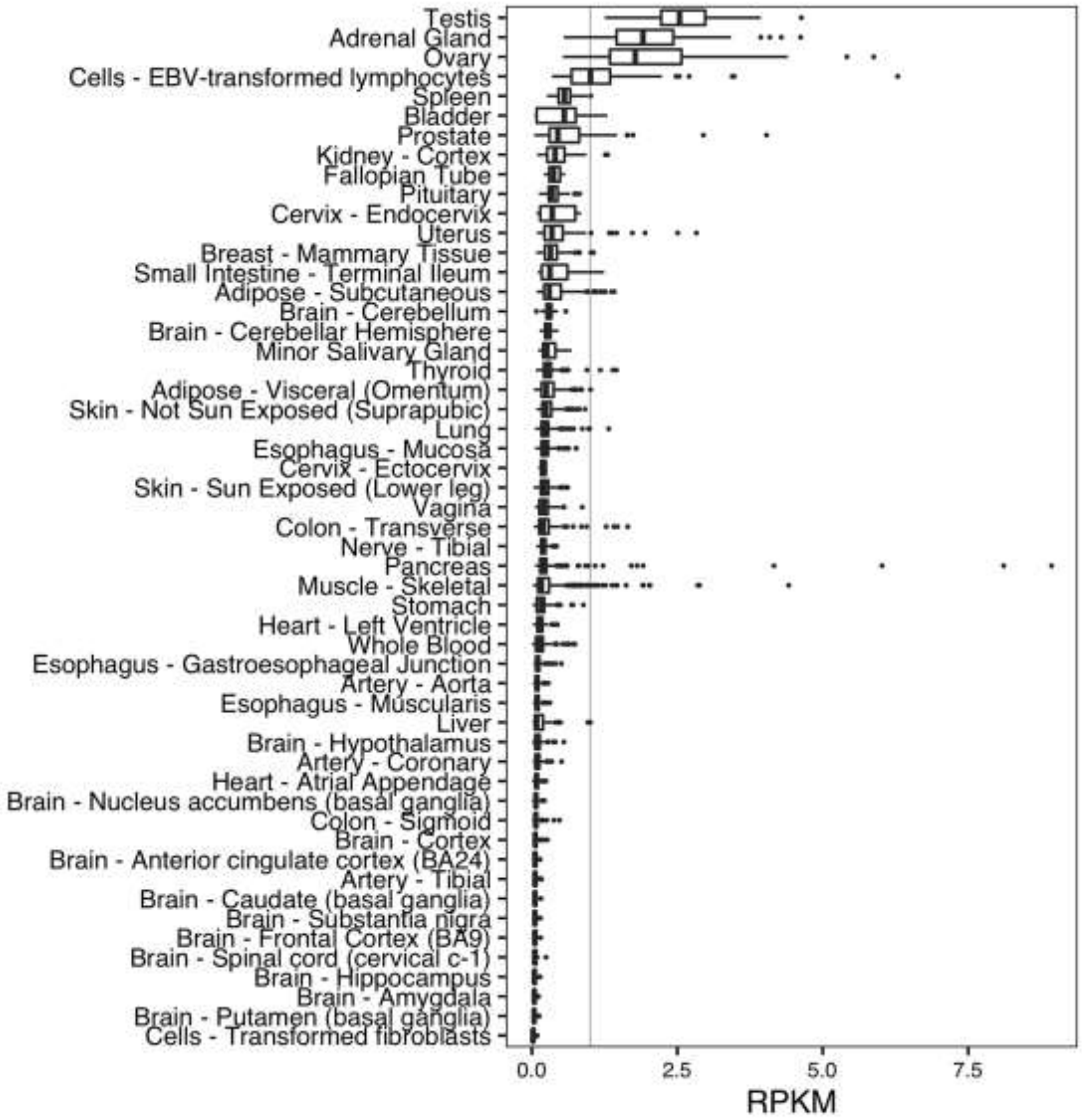

Supplementary Figure 3. Relevant levels of ERβ limited to few tissues according to GTEx data

Expression data from the Genotype-Tissue Expression (GTEx) consortium. 53 normal tissues from multiple individuals (n=5-357 patients/tissue) display ERβ transcript expression levels. A level of 1.0 read per kilobase and million (RPKM), indicated by line in figure, is unlikely to be present in the average cell nor result in protein. Tissues which display ERβ transcript levels of 1.0 RPKM or above are limited to testis (n=172), adrenal gland (n=145), ovary (n=97) and lymphocytes (EBV-transformed cells, n=118). Levels below 0.5 RPKM were noted for breast (n=214), adipose tissue (n=350), various areas of the brain (n=96-125), prostate (n=106), lung (n=320) and other.

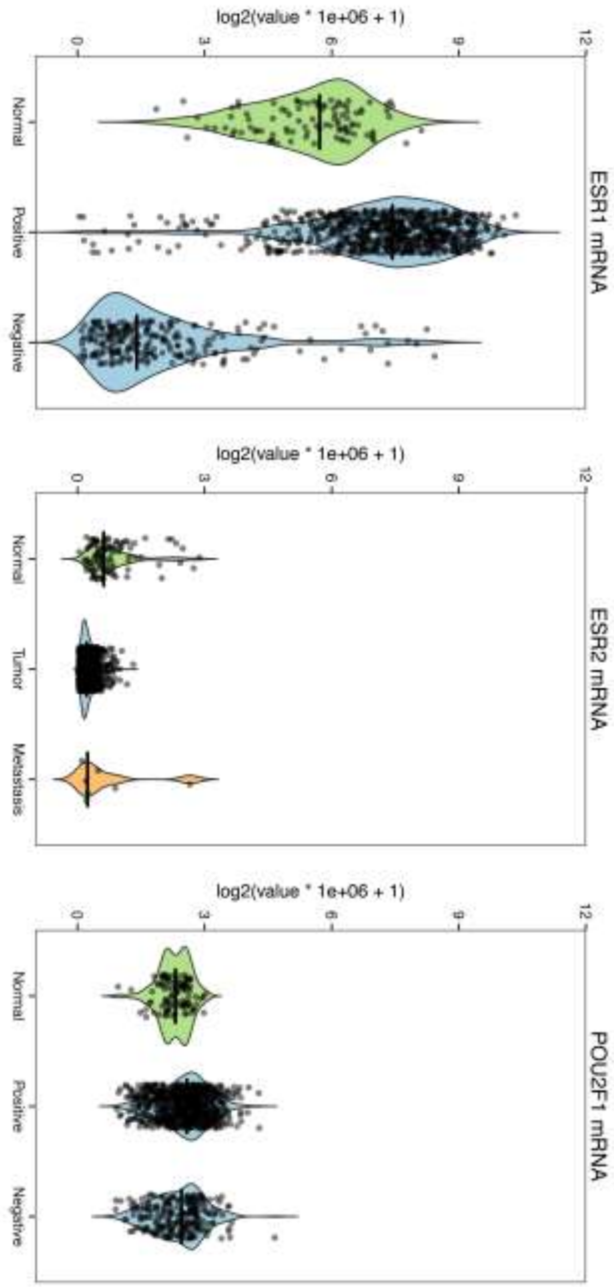

Supplementary Figure 4. RSEM expression estimates of ERα (ESR1), ERβ (ESR2) and POU2F1 in TCGA's BRCA cohort

RSEM expression estimates of transcript levels in 99 normal breast specimens, 995 breast tumors, and 7 distant metastases. For both ERα and POU2F1 expression graphs, the tumor samples are divided into ERα-positive and ERα-negative status based on clinical pathology diagnosis. For ERβ (middle) samples are divided into normal, tumor and metastatic samples. The violin plots show probability densities for the expression in the indicated subsets of the cohort, with the medians indicated by black crossbars.

Protein	Antibody	Species/clonality	Company	Catalogue # /Lot#	Dilution (IHC, optimized)
**ERα**	**1D5**	Mouse/mAb	DAKO	M7047 / 00077836	1:150
**ERβ**	**PPZ0506**	Mouse/mAb	Perseus Proteomics (Invitrogen, R&D)	417100/A-2	1:600
	**14C8**	Mouse/mAb	GeneTex	GTX70174/42142, 40784	1:1500
	**PPG5/10**	Mouse/mAb	DAKO<br>BioRad<br>Thermofisher	M7292 / 00076093<br>MCA1974G1 /161031<br>MA1-81281 / 74783060	1:60
	6A12	Mouse/mAb	Novus	NB200-303 / 1	1:225, 1:300
	ab133467	Rabbit/mAb	Abcam	ab133467 / GR97322-1	1:250
	68-4	Rabbit/mAb	Upstate (Millipore)	05-824 /n.d.	1:300
	ERb_503	Chicken/pAb	*In-house produced* [2]	*n/a*	1:900
	H150	Rabbit/pAb	Santa Cruz Biotechnology	sc-8974 / C1312	1:200
	N-terminal	Rabbit/pAb	*In-house produced* [3]	*n/a*	1:300
	ab137381	Rabbit/pAb	Abcam	ab137381 / GR106256-1	1:150
	CT	Rabbit/pAb	Upstate (Millipore)	07-359 /n.d.	1:200, 1:400
	HPA056644	Rabbit/pAb	*In-house produced (HPA)*	*n/a*	1:300
	ab3577	Rabbit/pAb	Abcam	ab3577 / GR73417-1	1:750

Supplementary Table 1. Antibodies included in the study
Antibodies evaluated using IHC in the Human Protein Atlas tissue collection are indicated in bold. Commercial antibodies include catalogue number and lot number, as available. Two antibodies were tested from more than one vendor (PPZ0506, PPG5/10). 'Dilution' indicates the optimized condition used for IHC on tissues.

**Cells**	**Cores/cell line**
T47D-ERβ (ERα+/ERβ+)	2
T47D-mock (ERα+/ERβ-)	2
HCT116-ERβ (ERα-/ERβ+)	2
HCT116-mock (ERα-/ERβ-)	2
**Tissues**	**No of patients/cores per patient**
Small intestine	2 / 2
Breast	2 / 2
Tonsil	2 / 2
Liver	2 / 2
Testis	2 / 2
Skeletal muscle	2 / 2
Colorectal cancer	2 / 2
Breast cancer (ERα+)	3 / 2
Breast cancer (ERα-)	2 / 2

Supplementary Table 2. Tissues and cells included in the ERβ tissue microarray (TMA) used for validation of antibodies

	Uniprot Accession	Name	Gene Name
1	P03372	ESR1_HUMAN Estrogen receptor	ESR1
2	P03372-2	ESR1_HUMAN Isoform 2 of Estrogen receptor	ESR1
3	P03372-3	ESR1_HUMAN Isoform 3 of Estrogen receptor	ESR1
4	P03372-4	ESR1_HUMAN Isoform 4 of Estrogen receptor	ESR1
5	Q92731	ESR2_HUMAN Estrogen receptor beta	ESR2
6	Q92731-2	ESR2_HUMAN Isoform 2 of Estrogen receptor beta	ESR2
7	Q92731-3	ESR2_HUMAN Isoform 3 of Estrogen receptor beta	ESR2
8	Q92731-4	ESR2_HUMAN Isoform 4 of Estrogen receptor beta	ESR2
9	Q92731-5	ESR2_HUMAN Isoform 5 of Estrogen receptor beta	ESR2
10	Q92731-6	ESR2_HUMAN Isoform 6 of Estrogen receptor beta	ESR2
11	Q92731-7	ESR2_HUMAN Isoform 7 of Estrogen receptor beta	ESR2
12	Q92731-8	ESR2_HUMAN Isoform 8 of Estrogen receptor beta	ESR2
13	G3V5S2	G3V5S2_HUMAN Estrogen receptor beta	ESR2
14	G3V5M5	G3V5M5_HUMAN Estrogen receptor beta	ESR2
15	F5GWJ8	F5GWJ8_HUMAN Estrogen receptor beta	ESR2
16	Q0PTK2	Q0PTK2_HUMAN Estrogen receptor beta 4	
17	Q59F41	Q59F41_HUMAN Estrogen receptor 2 (ER beta) variant (Fragment)	
18	Q99527	GPER1_HUMAN G-protein coupled estrogen receptor 1	GPER1
19	F1D8N3	F1D8N3_HUMAN Estrogen nuclear receptor beta variant b	NR3A2
20	P11474	ERR1_HUMAN Steroid hormone receptor ERR1	ESRRA
21	P11474-2	ERR1_HUMAN Isoform 2 of Steroid hormone receptor ERR1	ESRRA
22	O95718	ERR2_HUMAN Steroid hormone receptor ERR2	ESRRB
23	O95718-2	ERR2_HUMAN Isoform 2 of Steroid hormone receptor ERR2	ESRRB
24	O95718-3	ERR2_HUMAN Isoform 3 of Steroid hormone receptor ERR2	ESRRB
25	P62508	ERR3_HUMAN Estrogen-related receptor gamma	ESRRG
26	P62508-2	ERR3_HUMAN Isoform 2 of Estrogen-related receptor gamma	ESRRG
27	P62508-3	ERR3_HUMAN Isoform 3 of Estrogen-related receptor gamma	ESRRG
28	P62508-4	ERR3_HUMAN Isoform 4 of Estrogen-related receptor gamma	ESRRG
29	P62508-5	ERR3_HUMAN Isoform 5 of Estrogen-related receptor gamma	ESRRG

Supplementary Table 3. Proteins included in the in-house constructed FASTA library used for directed search in mass spectrometry analysis

Normal tissue types	No of patients/ cores per patient	Normal tissue types, cont.	No of patients/ cores per patient
Cerebral cortex	3 / 1	Ovary	3 / 1
Hippocampus	3 / 1	Endometrium	3 / 1
Nasopharynx	3 / 1	Cervix, uterine	3 / 1
Salivary gland	3 / 1	Vagina	3 / 1
Soft tissue	3 / 1	Urinary bladder	3 / 1
Bronchus	3 / 1	Bone marrow	3 / 1
Lung	3 / 1	Skin	3 / 1
Lymph node	3 / 1	Placenta	3 / 1
Liver	3 / 1	Pancreas	3 / 1
Adrenal gland	3 / 1	Fallopian tube	3 / 1
Gallbladder	3 / 1	Kidney	3 / 1
Duodenum	3 / 1	**Malignant tissue types**	**No of patients/ cores per patient**
Small intestine	3 / 1	Prostate cancer	12 / 2
Colon	3 / 1	Breast cancer	12 / 2
Appendix	3 / 1	Colorectal cancer	12 / 2
Smooth muscle	3 / 1	Ovarian cancer	12 / 2
Rectum	3 / 1	Cervical cancer	12 / 2
Seminal vesicle	3 / 1	Endometrial cancer	12 / 2
Prostate	3 / 1	Lymphoma	12 / 2
Epididymis	3 / 1	Glioma	12 / 2
Testis	3 / 1	Thyroid cancer	4 / 2
Skeletal muscle	3 / 1	Head and neck cancer	4 / 2
Lateral ventricle	3 / 1	Carcinoid	4 / 2
Cerebellum	3 / 1	Lung cancer	12 / 2
Oral mucosa	3 / 1	Melanoma	12 / 2
Tonsil	3 / 1	Skin cancer	12 / 2
Thyroid gland	3 / 1	Renal cancer	12 / 2
Parathyroid gland	3 / 1	Urothelial cancer	12 / 2
Esophagus	3 / 1	Testicular cancer	12 / 2
Heart muscle	3 / 1	Gastric cancer	12 / 2
Breast	3 / 1	Pancreatic cancer	12 / 2
Stomach	3 / 1	Liver cancer	12 / 2
Spleen	3 / 1	Granulosa cell cancer	12 / 2

Supplementary Table 4. Extended screening TMA used for tissue-wide detection of ERβ and ERα expression in normal and malignant tissues

Supplementary References

1. Uhlén, M., *et al.* Proteomics. Tissue-based map of the human proteome. *Science* **347**, 1260419 (2015).
2. Saji, S., *et al.* Estrogen receptors alpha and beta in the rodent mammary gland. *Proc Natl Acad Sci U S A.* **97**, 337-342 (2000).
3. Förster, C., Kietz, S., Hultenby, K., Warner, M. & Gustafsson, J.A. Characterization of the ERbeta-/-mouse heart. *Proc Natl Acad Sci U S A* **101**, 14234-14239. (2004).
